# Primary Psychiatric Disorder Masking the Diagnosis of Lupus Cerebritis

**DOI:** 10.7759/cureus.11643

**Published:** 2020-11-23

**Authors:** Waqas Memon, Zobia Aijaz, Muhammad S Afzal, Shujaa Faryad

**Affiliations:** 1 Internal Medicine/Nephrology, Virginia Commonwealth University, Richmond, USA; 2 Internal Medicine, Dow University of Health Sciences, Karachi, PAK; 3 Medicine, Carle Foundation Hospital, Champaign, USA; 4 Pulmonary/Critical Care, University of Illinois, Champaign, USA

**Keywords:** systemic lupus erythema, adult neurology, cns effects, clinical psychiatry

## Abstract

Systemic lupus erythematosus (SLE) is a chronic autoimmune disease that is known to affect different organs in the body. Nervous system involvement is common and can manifest as neurological or neuropsychiatric symptoms. A 23-year-old female with no significant past medical history, presented with nausea and vomiting for two weeks and unusual behavior for three days. Brain magnetic resonance imaging (MRI) showed small vessel ischemic changes and abnormal T2 flair/periventricular signal. Lab workup was positive for anti-dsDNA antibodies. The patient was diagnosed with SLE; positive serology and multisystem involvement including neurologic, serositis, and musculoskeletal system. Acute onset of abnormal behavior and memory problems were attributed to lupus cerebritis. The patient was started on methylprednisolone and had significant improvement in neurologic status within the next two days.

## Introduction

Systemic lupus erythematosus (SLE) is a chronic autoimmune disease that is known to affect different organs in the body. Nervous system involvement is common and can manifest as neurological or neuropsychiatric symptoms. Lupus cerebritis is the term used to describe neuropsychiatric manifestations of SLE. It can present as an acute confusional state, cognitive dysfunction, mood changes, lethargy, seizures, and coma. Lupus cerebritis can present during the course of the disease or even before the diagnosis. Cognitive dysfunction, a manifestation of lupus cerebritis, has been reported to occur in 20-80% of patients with SLE. Our patient had confusion and cognitive dysfunction (inability to do basic tasks, memory disruption) on the presentation without the existing diagnosis of SLE. Despite the frequent involvement of the nervous system, it remains a challenge to diagnose SLE based on neuropsychiatric or neurological manifestations especially if these are the initial presenting features of the disease as seen in our case. There is no definitive testing to confirm the diagnosis. Lupus cerebritis is the diagnosis of exclusion, as one needs to rule out the other potential causes including infections, electrolyte disturbances, mass lesions, and primary psychiatric disorders. High clinical suspicion is needed to reach the diagnosis and start treatment as timely intervention leads to improved outcomes.

## Case presentation

A 23-year-old female with a family history of SLE, presented with nausea and vomiting for two weeks and “not acting like herself” for three days. The patient had been admitted two weeks prior for calculus cholecystitis and underwent cholecystectomy. She was accompanied by a nurse caretaker, who said the patient had been acting like a child for the last three days. She had also been experiencing joint pain before hospitalization. On exam, she was normotensive with a blood pressure of 138/85, tachycardic with a heart rate of 106, and a temperature of 98.6°F. She was awake and alert with a Glasgow Coma Scale of 12. She was unable to recall recent events. The general examination was otherwise unremarkable.

**Figure 1:**
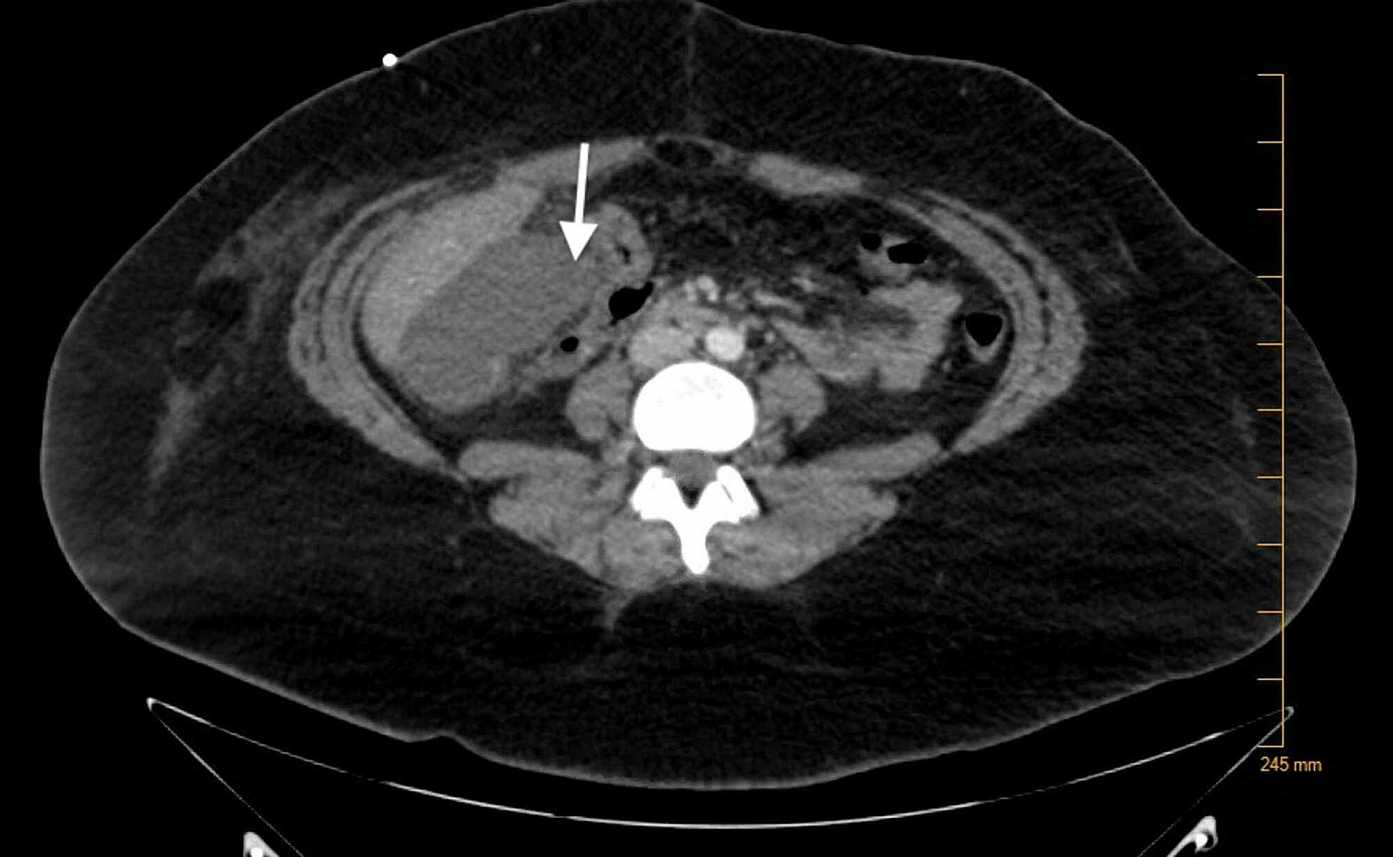
Fluid collection present in the gallbladder fossa indicating a post-operative seroma.

**Figure 2:**
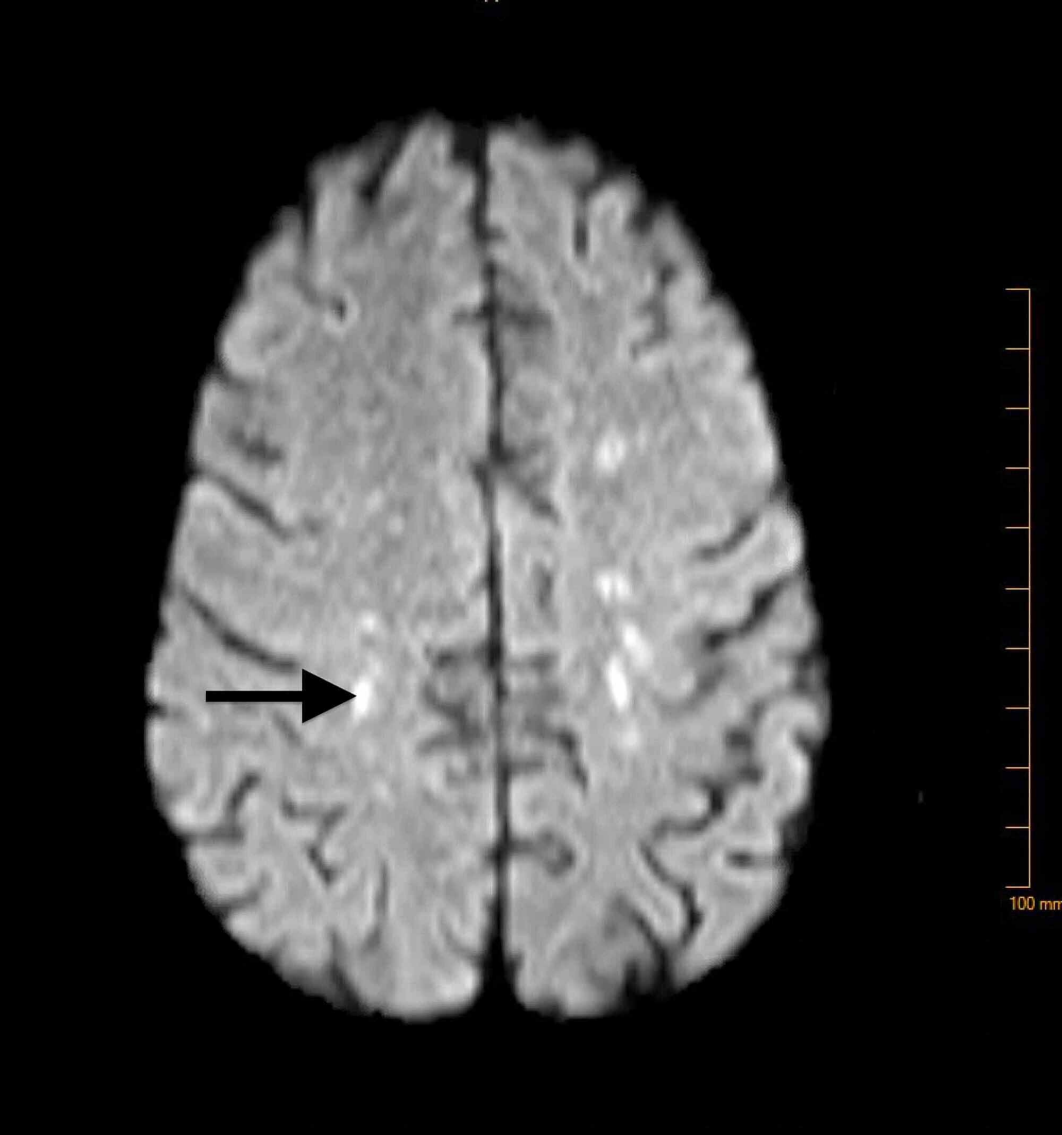
No evidence of acute infarction, multiple tiny foci of increased signal in the deep parietal white matter bilaterally.

**Figure 3:**
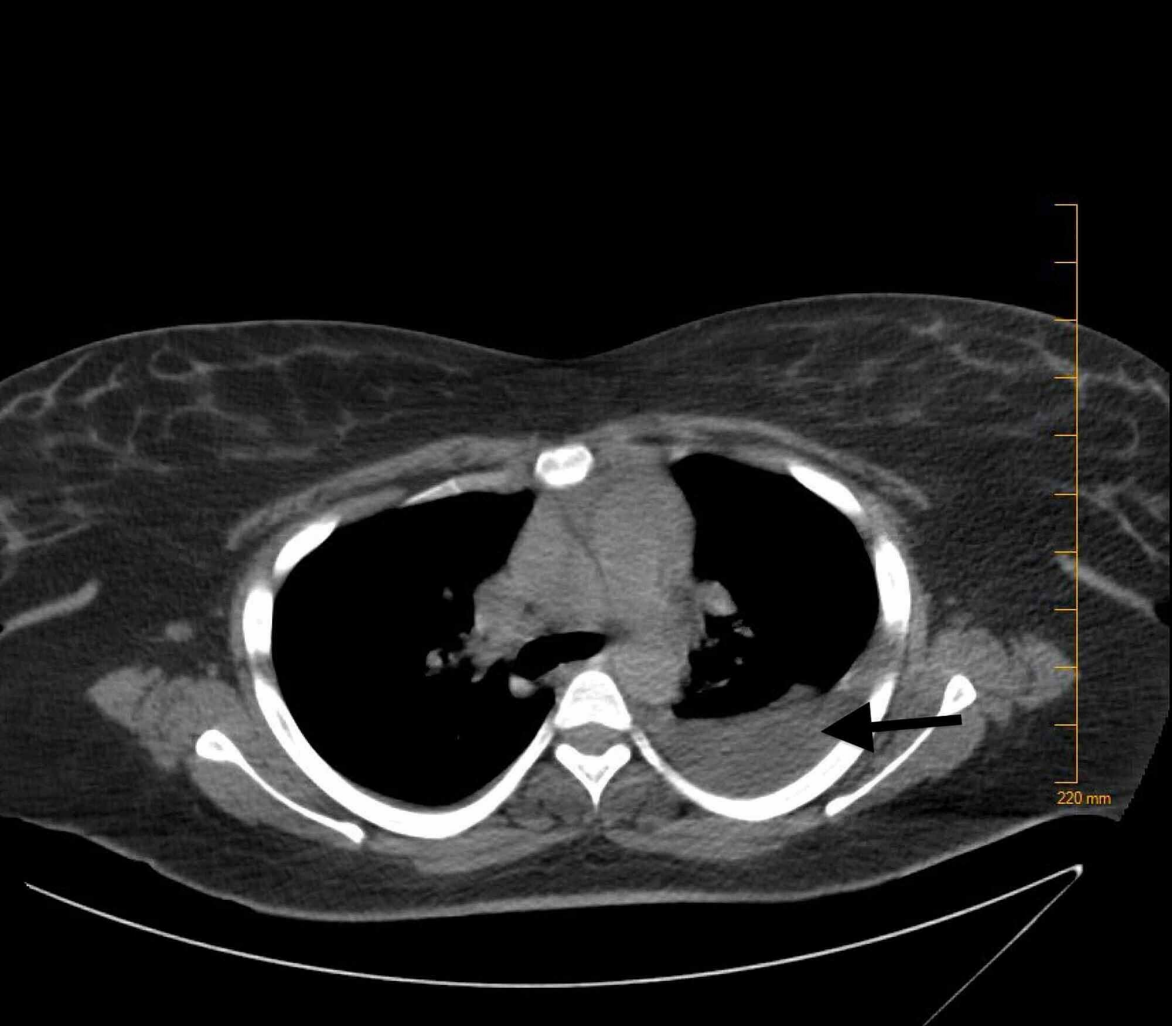
Moderate-sized pleural effusion on the left with left lower lung compression atelectasis. Mild right basilar atelectasis.

At the time of admission, complete blood count (CBC) revealed a hemoglobin of 7.8 g/dL (reference range: 12.0-15.8 g/dL), hematocrit of 24.1% (reference range: 36.0-47.0%), mean corpuscular volume (MCV) of 92.3 fL (reference range: 80-94 fL), mean corpuscular hemoglobin concentration (MCHC) of 32.7 g/dL (reference range: 33-37 g/dL). Complete metabolic panel (CMP) revealed hypokalemia of 2.9 mmol/L (reference range: 3.5-5.1 mmol/L), and total bilirubin of 1.2 mg/dL (reference range: 0.2-0.8 mg/dL). A computed tomography (CT) scan of the abdomen and pelvis was done. This revealed a fluid collection that was present in the gallbladder fossa suggesting a post-operative seroma (Figure 1). CT of the head (Figure 2) was done without contrast as the patient had a change in mental status, which did not show any acute intracranial abnormality. The patient continued to have confusion, meanwhile, a biliary drain was placed. During the admission, the patient also had some shortness of breath. A CT scan of the chest (Figure 3) was done and revealed a moderate-sized pleural effusion on the left, with compression atelectasis of the lower lung. There were also small calcified right hilar lymph nodes and subcarinal lymph nodes related to the old granulomatous disease. Small calcified right hilar lymph nodes and subcarinal lymph nodes related to old granulomatous disease were also appreciated.

**Figure 4:**
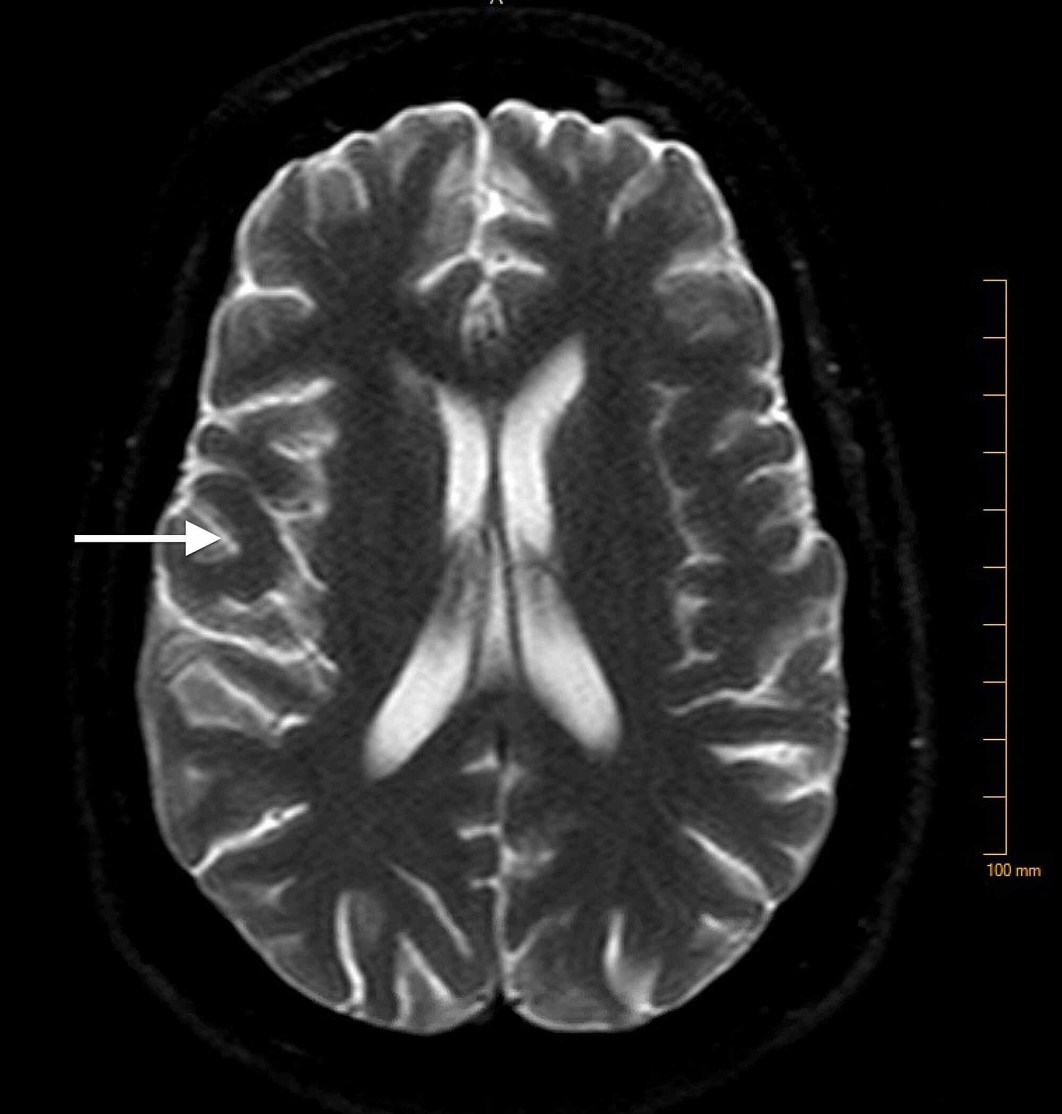
Small vessel ischemic changes and abnormal T2 flair/periventricular signal.

Neurological workup including lumbar puncture, brain MRI, and EEG was done. Brain MRI (Figure 4) showed small vessel ischemic changes and abnormal T2 flair/periventricular signal. EEG findings were consistent with diffuse cerebral dysfunction. Lumbar puncture findings were not significant for any pathology. Based on the MRI and EEG findings, the patient was suspected to have a multisystemic disorder and a rheumatologic workup was done. C-reactive protein (CRP) was 1.40 mg/dL (reference range <1.00 mg/dL), erythrocyte sedimentation rate (ESR) 47 mm/h (reference range: 0-20 mm/h), ANA titer >=1:2560, anti-double-stranded DNA Ab >300 IU/mL (reference range: <5 IU/mL), anti-Smith antibody 5.1 AI (reference range: <1.0 AI), anti-Smith/U1-RNP antibody 4.2 AI (reference range: <1.0 AI), positive perinuclear antineutrophil cytoplasmic antibody (pANCA).

## Discussion

SLE is an autoimmune condition, characterized by loss of self-tolerance with overactivation of cellular and humoral immune responses against self-antigens. The presentation features are variable ranging from inflammatory disease to critical renal, hematologic, or central nervous system involvement. Due to diverse presentation and resemblance to other autoimmune, infectious, or hematologic diseases, diagnosis is challenging particularly in the absence of pathognomic features. Although neuropsychiatric SLE is not uncommon its prevalence is estimated to be 10-80% [1-4]. Neuropsychiatric manifestations of SLE can be primary (related to disease activity) or secondary (related to complications of disease/treatment). Although secondary neuropsychiatric systemic lupus erythematosus (NPSLE) is common, primary NPSLE can be the initial presentation of SLE in patients such as similar in our case [5]. Neuropsychiatric symptoms of SLE can occur or flare-up while other symptoms are well controlled. The early identification of NPSLE can be challenging because it can present with negative serology and in the absence of systemic signs and symptoms [6]. Furthermore, neuropsychiatric manifestations in a patient with SLE may be secondary to comorbid conditions related to SLE, such as infection, severe hypertension, steroid psychosis and other drug toxicities, and metabolic abnormalities. Also, there may be a primary affective disorder unrelated to SLE. Regardless, central nervous system (CNS) involvement in SLE is associated with poor prognosis [5].

According to American College of Rheumatology (ACR), neuropsychiatric syndrome of SLE is divided into 12 central nervous system and seven peripheral nervous system manifestations. The central nervous system manifestations include cognitive dysfunction, delirium, psychosis, headache, cranial/peripheral neuropathy, and stroke. Less common are movement disorders, myelitis, and meningitis [7]. Cognitive dysfunction is an organic mental syndrome characterized by the presence of any one of the following symptoms: difficulty in short or long-term memory, language, executive function, impaired judgment and abstract thinking, and personality changes. It can range from stupor to mild agitation which contributes to difficulties in recognition. The profile of cognitive deficits seen in SLE is varied but the most frequently affected domains are attention, memory, visuospatial processing, language, problem-solving, speed of information processing, and executive function. In one previous similar case report, a young female patient presented with primary NPSLE with severe neurological symptoms due to cerebral vasculitis based on MRI findings and prompt treatment prevents the morbidity associated with it [8]. There are few reported cases where patients presented with primarily neurological presentation as the initial manifestation of SLE. Singh and Takács reported on a patient who developed cognitive decline, gait changes, and behavioral disturbances as the first symptoms of primary NPSLE [9]. Sommerlad et al. reported a case of acute confusional state and psychotic symptoms with a prolonged history of atypical seizure and cognitive decline [10]. The diagnosis was delayed due to atypical presentation and made by exclusion of other possible etiologies. In this clinical report, we describe an African-descendent female with symptoms of memory changes, confusion, and regression like behavior. These manifestations of NPSLE usually do not occur as the initial clinical presentation, leading to some difficult diagnostic challenges.

There are many bedside evaluation tools for the detection of cognitive impairment. The most common are MoCA (Montreal Cognitive Assessment), MMSE (Mini-mental state examination), Mini cog, the Ascertain dementia 8-word questionnaire, 5-word recall memory test. At present, no simple screening test for cognitive dysfunction is available for use in patients with SLE, as most such tests lack sensitivity for mild but clinically relevant dysfunction. After diagnosing cognitive impairment it is imperative to investigate for infections, medications, metabolic, neoplastic, and primary psychiatric disorders before attributing the symptoms to NSLE. The approach to patients with neuropsychiatric symptoms in SLE consists of investigations that first establish the diagnosis of SLE, distinguish between organic and functional conditions, and exclude further into non-SLE etiologies. Diagnosis is based on clinical assessment, immunoserologic testing, cerebrospinal fluid (CSF) analysis, electrophysiological studies, neuroimaging, and neuropsychological testing [11].

The predominant mechanism for primary CNS injury in lupus is microangiopathy arising from the increasing concentration of cytokines and complement system activation leading to microinfarction and cerebral ischemia [6,12]. Endothelial cells are responsible for the maintenance of the blood-brain barrier (BBB). Loss of integrity of the blood-brain barrier induced by different mechanisms, including infections and metabolic derangement, plays an important part in the pathophysiology of neuropsychiatric symptoms. Autoantibodies also induce cytokine productions including IL-6 and IL-8 that further cause damage to the BBB. Autoantibodies then enter the brain and cause neuronal damage [6,13]. The European League Against Rheumatism (EULAR)/ACR 2019 classification criteria for SLE requires a positive antinuclear antibody (ANA) as an obligatory entry criterion along with additive criteria in clinical and immunological domains. However non-SLE patients with symptoms such as headache and fatigue along with positive ANA titers can mimic NPSLE [14]. Therefore, serum levels of antibodies like anti-DNA and anti-Smith antibodies along with low levels of complement help in establishing the diagnosis of SLE. Anti-dsDNA antibodies correlate with other manifestations of SLE, especially glomerulonephritis but not with neuropsychiatric SLE. Once NPSLE is suspected, antibodies including APL, anti-ribosomal-P, anti-neuronal, and anti-NMDA antibodies can be useful for diagnosis [15]. Karassa et al. conducted the meta-analysis to determine the association of anti-ribosomal-P and NPSLE, concluding that it has limited diagnostic value and cannot differentiate between different subgroups of NPSLE [16]. However, Wang et al. have concluded that anti-P antibodies are more valuable in the diagnosis of SLE in the absence of specific antibodies and associated with an earlier age of onset [17].

The role of APL antibodies demonstrated a significant association with neuropsychiatric manifestation, especially with cerebrovascular disease. Kivity et al. have reported that antiphospholipid antibody (APLA), anti-N-methyl-D-aspartate receptor (NMDA), and anti-GABA may be related to many neuropsychiatric manifestations, including cognitive dysfunction [6]. Some studies have found a relationship between antiphospholipid antibodies and thrombotic events, such as cerebrovascular disease and microangiopathy. This further affects the vascularization of the brain resulting in cognitive decline and function [18]. Therefore, given the numerous manifestations of NPSLE and studies reporting associations, none of these autoantibodies can be concluded to be a definite biomarker of NPSLE. Goswami et al. reported two cases where CSF analysis in a patient with neuropsychiatric SLE reveals pleocytosis with cell count <500 and lymphocytic predominance with elevated CSF protein, IL-6, IFN- alpha levels [19]. However, CSF studies are useful to differentiate between infectious and NPSLE, it is not always required for the management of NPSLE [20]. In this case, lumbar puncture is performed due to atypical presentation and uncertain diagnosis. The results of an analysis of cerebrospinal fluid were within the normal range, which is not unusual in the setting of NPSLE.

Electroencephalography is primarily used to investigate seizure disorders, it usually detects asymmetry of the electric cerebral activity and diffuse disorganized background activity in the scenario of cognitive dysfunction. Neuroimaging studies can identify changes caused by SLE and further exclude the alternative diagnosis. MRI is the investigation of choice and frequently detects lacunar infarcts, parenchymal atrophy, and white matter lesions and has been correlated with the severity of cognitive dysfunction.

According to EULAR (2014) report for NPSLE, MRI is indicated if the patient is less than 60 years of age, cognitive dysfunction is severe and rapidly evolving and development of cognitive dysfunction in the setting of immunosuppressive or antiplatelet/anticoagulation therapy, onset of other NPSLE symptoms, and recent and significant head trauma. Advanced MRI modalities and functional neuroimaging are reserved in cases when conventional MRI findings are normal or inconclusive. Functional impairment due to severe cognitive dysfunction should be confirmed by a battery of neuropsychological tests proposed by the ACR. If abnormalities are detected by the use of these tests, repeat testing using the same battery should be done after an interval for the follow-up to further assess the performance after treatment. The computer-assisted neuropsychological assessment also facilitates the efficient screening of patients with cognitive difficulties. However, these tests require specialized training, are time-consuming, and are subject to practice effects with repeated use [11]. This neuropsychological testing was not done in this case but may have diagnostic clue. We concluded that in our case diagnosis of active NPSLE was based on combining the presence of serositis (pleural effusion), anemia, imaging findings with high ANA, and anti-dsDNA levels but negative APLA. The absence of meningeal signs and pathological reflexes made meningitis or encephalitis unlikely. However, nutritional deficiencies have been well recognized to cause severe confusion, it is unlikely in our case since the patient was not able to improve even after the treatment with replacement therapy.

Management of CNS involvement is crucial and requires close monitoring of disease activity with frequent adjustments in therapy. Treatment of NPSLE includes therapy targeted at an underlying mechanism like autoantibody-mediated damage or a hypercoagulable state with symptomatic management such as anti-epileptic, anti-depressive, anti-neuropathy, and other medications. In 2019, a European League Against Rheumatism (EULAR) update recommended that all patients with SLE should be started on hydroxychloroquine. Systemic glucocorticoids are most commonly used and they are effective in up to 75% of patients such as in this case. The usual regimen for severe disease is initial IV pulse therapy for 3-5 days followed by oral therapy. In a patient with severe disease additional immunosuppressive therapies such as methotrexate, cyclophosphamide, hydroxychloroquine, azathioprine, have also been used and they allow glucocorticoids taper off as well. Cyclophosphamide is favorable in severe and refractory diseases such as neuro SLE, cardiopulmonary, and renal but not recommended in women of reproductive age. In a patient with refractory disease, rituximab, IVIG, and plasma exchange can be considered as well [14]. Our patient initially received intravenous methylprednisolone in high dose after the diagnosis and made recovery with a good response to treatment. She was discharged on prednisone and was later started on hydroxychloroquine and was tapered off the glucocorticoids. Cyclophosphamide due to its gonadotoxic effects was not preferred in our case. Psycho-educational group interventions should be considered as these have demonstrated improvements in memory and activities of daily living.

## Conclusions

The patient was diagnosed with SLE; positive serology and multisystem involvement including neurologic, serositis, and musculoskeletal system. Acute onset of abnormal behavior and memory problems were attributed to lupus cerebritis. The patient was started on methylprednisolone and had significant improvement in neurologic status within the next two days.
